# Validation of the Chinese Version of KIDSCREEN-10 Quality of Life Questionnaire: A Rasch Model Estimation

**DOI:** 10.3389/fpsyg.2021.647692

**Published:** 2021-08-16

**Authors:** Zepeng Gong, Jia Xue, Ziqiang Han, Yuhuan Li

**Affiliations:** ^1^School of Public Affairs and Administration & Shenzhen Institute for Advanced Study, University of Electronic Science and Technology of China, Chengdu, China; ^2^Factor-Inwentash Faculty of Social Work & Faculty of Information, University of Toronto, Toronto, ON, Canada; ^3^School of Political Science and Public Administration, Shandong University, Qingdao, China; ^4^School of Government, Central University of Finance and Economics, Beijing, China

**Keywords:** quality of life, KIDSCREEN-10, psychometric property, Rasch analysis, China

## Abstract

The KIDSCREEN-10 was deemed as a cross-national instrument for measuring Health-Related Quality of Life (HRQoL). However, no empirical endeavor has explored its reliability and validity in the context of China. This study aims to translate and validate the Chinese version of the KIDSCREEN-10 questionnaire. The KIDSCREEN-10 was translated into Chinese (Mandarin) using a blindly bilingual forward–backward–forward technique. A cross-sectional survey, including 1,830 students aged from 8 to 18 years, was conducted in a county located in Gansu province, China. Psychometric properties were evaluated using the Rasch partial credit model, ANOVA, and the correlation analysis. Results indicated that the KIDSCREEN-10 performed good internal consistency, known-group validity, and concurrent validity, but there were still some deficiencies in psychometrics: first, disordered response categories were found between category 2 (seldom) and category 3 (sometimes); second, item 3 (“Have you felt sad?”), item 4 (“Have you felt lonely?”), and item 5 (“Have enough time for self?”) demonstrated misfit to the Rasch model; third, items 3 and 4 exhibited differential item functioning. After collapsing the disordered response categories and removing the three misfit items, the seven-item questionnaire performed good psychometric properties. However, the seven-item version does not cover the psychological well-being dimension of HRQoL, and that may lead to inappropriate measures of HRQoL. Therefore, this paper suggested to use classical test theory to investigate the psychological properties of the KIDSCREEN-10.

## Introduction

Quality of life (QoL) is an important public health issue for the policy development (Phillips, 2006). QoL refers to the perception of subjective health and well-being of an individual, which is a multidimensional concept covering several dimensions, including social relationships, physical and psychological health (The World Health Organization, 1995). Assessing the QoL more scientifically is an essential step to improve QoL of people's, and researchers have invested lots of effort into developing appropriate assessment tools for the general public or specific groups. For example, there are generic- and condition-specific instruments for evaluating QoL of children and adolescents. The former one is applicable to all population subgroups, whereas the latter one is useful to those with specific disability or illness (Fava et al., 2009; Davis et al., 2013; Bullinger et al., 2015). This study focused on the generic QoL instrument for children and adolescents.

Currently, the KIDSCREEN (The KIDSCREEN Group Europe, 2006), the Pediatric Quality of Life Inventory 4.0 (PedsQL 4.0) (Varni et al., 2006), the KINDL (Ravens-Sieberer and Bullinger, 1998), the Child Health Questionnaire (CHQ) (Raat et al., 2002), and Brief Multidimensional Students' Life Satisfaction Scale (BMSLSS) (Huebner, 1994) are well-known generic questionnaires that have been adopted to assess QoL in children and adolescents. These measures share some commonalities, but each one has its preference. The KIDSCREEN was designed to measure health-related quality of life (HRQoL), the PedsQL 4.0 included a wide definition of functioning, disability, and health (FDH), the KINDL, and the CHQ were appropriate to evaluate FDH with some HRQoL features, and the BMSLSS focused on life satisfaction (Seligson et al., 2003; Alamolhoda et al., 2021). To our knowledge, psychometric properties of the PedsQL 4.0, the KNIDL, the CHQ, and the BMSLSS have been validated in China (Ng et al., 2005; Lin et al., 2012, 2014; Ye et al., 2014), where has a population of 321 million children and adolescents under 20 years old (National Bureau of Statistics, 2011), whereas the KIDSCREEN has not been fully tested in this context.

KIDSCREEN instruments include self-report and proxy (parents) versions, and each of the two versions has three forms with 52, 27, and 10 items, respectively. The tools were initially and simultaneously developed in 13 European countries (The KIDSCREEN Group Europe, 2006). The KIDSCREEN-52 instrument with 52 items assesses 10 dimensions of HRQoL: physical well-being, psychological well-being, moods and emotions, self-perception, autonomy, parent relation and home life, financial resources, peers and social support, school environment, and bullying (Ravens-Sieberer et al., 2008; Zhu et al., 2019). The KIDSCREEN-27 is a short version of KIDSCREEN-52 using 27 items to measure five facets (i.e., physical well-being, psychological well-being, autonomy and parent relation, social support and peers, and school environment) merged from the 10 dimensions mentioned above (Ng et al., 2015). The KIDSCREEN-10 comprises 10 items derived from the 27-item version (The KIDSCREEN Group Europe, 2006). Evidence from prior studies indicated that KIDSCREEN-10 results in one global HRQoL score (Ravens-Sieberer et al., 2010; Haraldstad et al., 2011).

The KIDSCREEN instruments are deemed as cross-national HRQoL measures, and their psychometric properties have been studied in considerable research. Ravens-Sieberer et al. have conducted a cross-cultural survey in 13 European countries to assess the reliability and validity of KIDSCREEN indexes, and the result showed that all three versions of KIDSCREEN were reliable and valid (Robitail et al., 2007; Ravens-Sieberer et al., 2008, Ravens-Sieberer et al., 2010). Moreover, such supportive result for KIDSCREEN-52 has been found in investigations from China (Ng et al., 2015; Zhu et al., 2019), South Korea (Hong et al., 2007), South Africa (Taliep and Florence, 2012), Serbia (Stevanovic et al., 2013), Iran (Parizi et al., 2014), Japan (Nezu et al., 2015), Turkey (Baydur et al., 2016), and Colombia (Jaimes-Valencia et al., 2019, 52). In addition, studies from China (Ng et al., 2015), Serbia (Stevanovic et al., 2013), Turkey (Baydur et al., 2016), Norway (Andersen et al., 2016), Japan (Nezu et al., 2016), and Colombia (Vélez et al., 2016) have found a similar result for KIDSCREEN-27. Regarding KIDSCREEN-10, its validity and reliability can be supported in studies from Serbia (Stevanovic et al., 2013), Turkey (Baydur et al., 2016), Japan (Nezu et al., 2016), and Iran (Nik-Azin et al., 2014). Comparatively, a little evidence evaluated measurement properties of KIDSCREEN-10, especially no empirical endeavor has explored its reliability and validity in China. Given KIDSCREEN-10 was recommended for large epidemiological studies (The KIDSCREEN Group Europe, 2006), it is necessary to translate and test the applicability of KIDSCREEN-10 in more countries.

The current study aims to validate the cross-cultural adaption of the Mandarin Chinese self-report questionnaire of KIDSCREEN-10 using the Rasch model. Although the KIDSCREEN-52 and the KIDSCREEN-27 have been tested in the context of China (Ng et al., 2015; Zhu et al., 2019), we cannot infer that the KIDSCREEN-10 originally developed them also has good psychometric properties. For example, the KIDSCREEN-52 and the KIDSCREEN-27 are multidimensional scales, whereas the KIDSCREEN-10 is deemed as an unidimensional measure (Ravens-Sieberer et al., 2014). Therefore, based on the results of Zhu et al. (2019) and Ng et al. (2015), we still do not know whether the KIDSCREEN-10 is unidimensional. Moreover, the Rasch model is an approach exploring the performance of each item rather than the total test score, as in the classical test theory (CTT) (da Rocha et al., 2013). The Rasch model provides a detailed analysis of how items work within scales (Tennant et al., 2004), and thus it has many potential advantages over CTT methods in evaluating self-reported health outcomes (Hays et al., 2000). Currently, it has been increasingly applied in the psychology and health fields (Rocha et al., 2012; Ng et al., 2015; Vélez et al., 2016). Previous studies have demonstrated that this approach is appropriate, and actually more accurate to discover the psychometric properties of KIDSCREEN-10 (Erhart et al., 2009; Ravens-Sieberer et al., 2014). Therefore, the Rasch model is adopted to examine the properties of the KIDSCREEN-10 instrument for measuring the HRQoL among Chinese children and adolescents.

## Methods

### Sampling and Participants

We conducted a survey in students of all grades of middle, high, vocational schools and in Grade four to Grade six students of primary schools in a county of Gansu province, China. All participants completed the online questionnaires in the computer room of their schools with the help of research assistants and teachers. In total, 2,155 students participated our survey. After dropped those with missing value or aged above 18 years, 1,830 eligible respondents were obtained. Of the respondents, 50.98% were girls, and 38.31% were 8–12 years old and 61.69% aged from 12 to 18 years (Table 1). Students from primary, middle, high, and vocational schools accounted for 39.40, 33.17, 19.23, and 8.20%, respectively. Regarding ethnic minority group differences, a total of 660 students were Han (the national majority in China), 521 were Yugur, 568 were Tibetan, and 81 were other ethnic minorities, including the Uygur, Mongolian, Hui, and others.

**Table 1:** Sample characteristics.

	**Frequency**	**Percentage**
Gender		
Female	933	50.98
Male	897	49.02
Ethnicity		
Minorities	1,170	63.93
Majority	660	36.07
Age		
8–12 years	701	38.31
13–18 years	1,129	61.69
School type		
Primary school	721	39.40
Middle school	607	33.17
High school	352	19.23
Vocational school^+^	150	8.20
Socioeconomic status		
Low	16	0.87
Medium to low	149	8.14
Medium	1,043	56.99
High to medium	565	30.87
High	57	3.11
Health status		
Very bad	7	0.38
Bad	12	0.66
Moderate	164	8.96
Good	831	45.41
Very good	816	44.59
Academic performance		
Very bad	71	3.88
Bad	233	12.73
Moderate	694	37.92
Good	674	36.83
Very good	158	8.63

+ *vocational school is equivalent to high school but focusing on working skills training*.

### Instruments

#### KIDSCREEN-10

As mentioned above, KIDSCREEN-10 is a 10-item self-report questionnaire (Table 2). The same five-point Likert scale measures each item. In the current study, participants were asked: “In the last week, how often do you experience the following items?” The answers to each item were: never (score = 1), seldom (score = 2), sometimes (score = 3), often (score = 4), and always (score = 5). The total score of all the 10 items was calculated to assess HRQoL. A higher total score indicates a higher level of HRQoL. In order to ensure that Chinese students easily understand each item, the Mandarin Chinese version of KIDSCREEN-10 was translated from the English version using a blindly bilingual forward–backward–forward technique (Supplementary Table 1) (Brislin, 1970). First, two graduate students with good English and Chinese language skills translated the English version of KIDSCREEN-10 into Chinese (Mandarin) independently, and then they discussed their translation results with the supervisor and reached a consensus. Second, the Chinese version was given to another two graduate students for back-translation independently, and they also discussed and reached a consensus. Finally, a bilingual expert read and checked all the translation documents and confirmed the final version. The Mandarin KIDSCREEN-10 questionnaire requires ~3–5 min to complete.

**Table 2:** Items characteristics.

**Items**	**Mean**	**SD**	**Floor (%)**	**Ceiling (%)**
Item 1: felt fit and well	3.82	1.29	10.16	38.85
Item 2: felt full of energy	3.68	1.31	10.71	33.83
Item 3: felt sad	2.22	1.14	30.93	6.23
Item 4: felt lonely	2.09	1.18	39.18	6.39
Item 5: had enough time for self	2.89	1.30	16.89	15.08
Item 6: did things in free time	3.58	1.26	9.18	29.02
Item 7: treated fairly by parent(s)	3.94	1.29	9.07	46.17
Item 8: had fun with friends	4.11	1.15	6.56	48.03
Item 9: got on well at school	3.68	1.12	5.74	27.16
Item 10: pay attention	3.71	1.15	6.45	28.03

#### Brief Multidimensional Students' Life Satisfaction Scale

The BMSLSS was designed to assess the satisfaction of five life domains (i.e., family, friends, school, self, and environment) of students aged from 8 to 18 years (Seligson et al., 2003). In the present study, participants were asked to evaluate how satisfied with the five areas mentioned above. The responses to each area were ranged from “extremely dissatisfied (score = 1)” to “extremely satisfied (score = 5).” The participant with a higher total score calculated by summing the scores of five areas indicated he/she had a higher life satisfaction. The BMSLSS has been proved to have good psychometric properties in the context of China in prior studies (Ye et al., 2014; Tian et al., 2015). The Cronbach's alpha of BMSLSS in this study was 0.95.

#### Other Variables

Socio-demographics, including gender, ethnicity, and age, were investigated. Moreover, students reported that they perceived socioeconomic status, health status, and academic performance. Socioeconomic status was measured from the question, “Compared with other families in your living region, how do you think of your family's socioeconomic level?” Of the respondents, more than half of them perceived the economic status of their families were medium, and nearly one-third perceived that their status was above medium (Table 1). Regarding health status and academic performance, two questions, “how do you think of your health/academic performance?” were asked. Answers for these two questions were: very bad, bad, moderate, good, and very good. Most of the participants perceived their health as good or very good, and perceived their academic performance as moderate or above.

### Statistical Analysis

#### Descriptive Analysis

First, the frequency and percentage of socio-demographic variables were reported. Then, the mean score (SD) and distribution of the 10 items of KIDSCREEN-10 were examined. A floor or ceiling effects were considered significant if more than 20% of the participants responded to the item with the answer of never or always (Holmes and Shea, 1997).

#### Rasch Analysis

The Rasch partial credit model for ordered response categories was adopted to evaluate the measurement properties of KIDSCREEN-10 by using the WINSTEPS 4.0.1 software (Beaverton, Oregon: Winsteps.com). In this study, we evaluated six aspects of the scale: response format, item fit, person reliability, unidimensionality, item difficulty, and differential item functioning (DIF). First, we tested the appropriateness of the response format of KIDSCREEN-10 by checking the order of step difficulty. Step difficulty refers to the threshold between adjacent response categories of an item (Linacre, 1999). The response format (never = 1, seldom = 2, sometimes = 3, often = 4, and always = 5) was considered to be appropriate when the step difficulties were properly ordered (i.e., no disorder). If any disorder was detected between response categories, we merged the disordered categories and reexamined the step difficulty of response categories to obtain an appropriate response format. Second, infit mean square (MNSQ) and outfit MNSQ were used to evaluate item fit (i.e., data-model fit). The ideal value of infit and outfit MNSQ is 1, and the acceptable range of MNSQ value was set as 0.6 to 1.4 (Wong et al., 2011; Jervaeus et al., 2013). If the MNSQ value of any item exhibited unacceptable, we deleted the item and reconducted the analysis. Third, person-separation reliability was computed to estimate the internal consistency of KIDSCREEN-10. A reliability value >0.7 was considered to be adequate (Vélez et al., 2016). Fourth, unidimensionality means that all items in an instrument measure the same latent trait. To examine the KIDSCREEN-10 is unidimensionality, principal component analysis of residuals was conducted. The assumption of unidimensionality was considered to be acceptable if the eigenvalues of the first contrast was <2 (Wu et al., 2016). Finally, DIF reflects the potential bias of items understood by different groups. We should ensure that the response of participants to items based on the latent trait of interest regardless of other characteristics, such as gender or age (Baylor et al., 2011). Therefore, a well-performing item should not demonstrate DIF, i.e., one group of participants understand one item in the same way as another group of participants (Tennant et al., 2004). This study tested the DIF across gender (boys/girls), ethnicity (majority/minorities), and age (8–12 years/13–18 years). The DIF was detected when the absolute value of DIF contrast (the difference in the difficulty of an item between two groups) was >0.5 logits, and the *p*-value of Welch's test was lower than 0.05 (Bond et al., 2015).

#### Known-Group Validity

The known-group validity of KIDSCREEN-10 was assessed by comparing the discrepancy of HRQoL level between groups, such as different socioeconomic statuses, health statuses, and academic performance groups. One-way ANOVA was applied to examine the differences between groups. Eta square (η^2^) was computed. We considered the effect size magnitudes of η^2^ 0.01 as small, 0.06 as moderate, and 0.14 as large (Zhu et al., 2019).

#### Concurrent Validity

The Pearson correlation coefficients between KIDSCREEN-10 scores and BMSLSS scores were calculated to evaluate the concurrent validity of KIDSCREEN-10. The coefficient intervals of 0.1–0.3, 0.3–0.5, and 0.5 or more were considered as low, moderate, and large, respectively (Wu et al., 2013; Zhu et al., 2019).

## Results

### Item Characteristics

Characteristics of items are shown in Table 2. Item 8 had the largest mean value of 4.11, with a SD of 1.15. The mean value of other items ranged from 2.09 to 3.94. Additionally, floor effects were observed in item 3 and item 4, whereas other items demonstrated ceiling effects except item 5.

### Response Format

**Figure 1:**
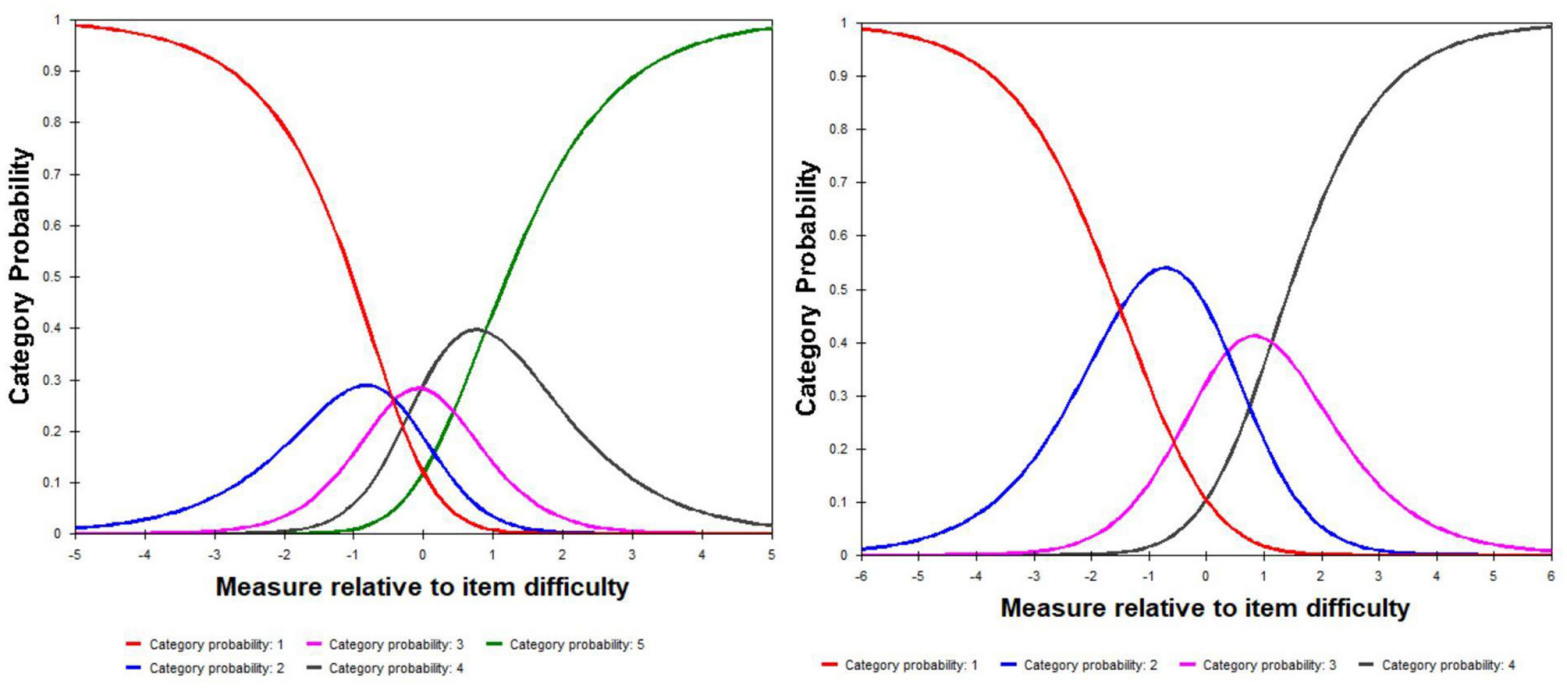
Category probability curves of item 2. The left figure was drawn based on the original five-point response categories (never = 1, rarely = 2, sometimes = 3, often = 4, always = 5); the right figure was drawn after merged the original category 2 (rarely) and 3 (sometimes), namely, the new response categories were 1 = never, 2 = rarely/sometimes, 3 = often, 4 = always.

Figure 1 shows the results of the step difficulty between adjacent response categories for item 2. There was category disorder in the left figure, which was drawn based on the original five-point response categories. After we collapsed the category 2 (seldom) and 3 (sometimes), then we reconducted Rasch analysis. The results from the right figure exhibited that no disorder presented in step difficulty.

### Item Fit

The results of the fit analysis are shown in Table 3. In this study, three versions of the questionnaire were analyzed to fit the Rasch model. The version 1 was the original KIDSCREEN-10 with a five-point measurement scale. As we can see, infit (outfit) MNSQ value for item 3 and item 4 were 1.59 (1.72) and 1.58 (2.00), respectively. According to the results of the response format analysis, we should merge the response category 2 and category 3. Thus, the version 2 was the KIDSCREEN-10 using a four-point scale. Similarly, item 3 (outfit MNSQ = 1.62) and item 4 (outfit MNSQ = 1.91) did not fit the expectation of the Rasch model. Then, we removed these misfit items and reconducted analysis and found that item 5 was also a misfit item (infit MNSQ = 1.62, outfit MNSQ = 1.59). Accordingly, three items (i.e., item 3/4/5) were dropped from the KIDSCREEN-10. The version 3 was the questionnaire without any misfit item. It showed that the total fit (mean of infit MNSQ = 1.00, mean of outfit MNSQ = 1.01) of items was adequate. In addition, infit and outfit values of all items demonstrated acceptable fitness to the Rasch model.

**Table 3:** Item difficulty and item fit statistics for KIDSCREEN before and after merged response categories 2 and 3.

	**Version 1**				**Version 2**				**Version 3**			
	**Item** **difficulty**	**Model** **SE**	**Infit** **MNSQ**	**Outfit** **MNSQ**	**Item** **difficulty**	**Model** **SE**	**Infit** **MNSQ**	**Outfit** **MNSQ**	**Item** **difficulty**	**Model** **SE**	**Infit** **MNSQ**	**Outfit** **MNSQ**
Item 1	−0.35	0.03	0.87	0.92	−0.48	0.03	0.83	0.88	0.09	0.04	1.00	1.04
Item 2	−0.23	0.03	0.77	0.79	−0.31	0.03	0.76	0.77	0.35	0.04	0.92	0.93
Item 3	1.28	0.03	1.59	1.72	1.67	0.04	1.58	1.62				
Item 4	1.37	0.03	1.58	2.00	1.79	0.04	1.62	1.91				
Item 5	0.48	0.02	1.00	1.02	0.63	0.03	1.02	1.01				
Item 6	−0.22	0.03	0.87	0.86	−0.28	0.03	0.86	0.85	0.45	0.04	1.30	1.33
Item 7	−0.49	0.03	0.92	0.90	−0.68	0.03	0.90	0.89	−0.20	0.04	1.10	1.15
Item 8	−0.80	0.03	0.81	0.77	−1.06	0.04	0.79	0.75	−0.70	0.04	0.86	0.84
Item 9	−0.55	0.03	0.79	0.79	−0.67	0.04	0.77	0.77	−0.02	0.04	0.94	0.94
Item 10	−0.48	0.03	0.77	0.78	−0.60	0.04	0.76	0.77	0.05	0.04	0.88	0.87
Mean	0.00	0.03	1.00	1.05	0.00	0.04	0.99	1.02	0.00	0.04	1.00	1.01
SD	0.73	0.00	0.30	0.41	0.96	0.00	0.32	0.39	0.35	0.00	0.14	0.16

*Version 1 is the KIDSCREEN-10 with 10 items measured by five-point scale; version 2 includes 10 items measured by four-point scale (after merged response categories 2 and 3); and version 3 includes seven items measured by four-point scale; MNSQ: mean square; standard error (SE)*.

### Reliability, Unidimensionality, and Item Difficulty

The person separation reliability value for version 1, 2, and 3 were 0.82, 0.85, and 0.83, respectively. These results reflected good internal consistency for three versions of the KIDSCREEN questionnaire. Regarding the unidimensionality, eigenvalue of the first contrast was 3.13 in the version 1, 2.91 in the version 2, and 1.98 in the version 3. Thus, only the version 3 exhibited unidimensionality. Moreover, in both version 1 and version 2, item 8 (“Have you had fun with your friends?”) was the easiest item, whereas item 4 (“Have you felt lonely?”) was the most difficult item (Table 3). In the version 3, the easiest and most difficult items were item 8 (difficulty value = −0.70) and item 6 (difficulty value = 0.45), respectively.

### Differential Item Functioning

The results of DIF tests are shown in Table 4. All items did not demonstrate DIF when we compared boys with girls, as well as the ethnicity of the majority with minorities. However, DIF was observed when comparing participants aged 8–12 years with 13–18 years, namely, item 4 (“have you felt lonely?”) exhibited DIF in version 1 (DIF contrast value = 0.56, *p* < 0.001) and version 2 (DIF contrast value = 0.86, *p* < 0.001), whereas item 3 (“have you felt sad?” in version 2 (DIF contrast value = 0.68, *p* < 0.001).

**Table 4:** Differential item functioning by gender, ethnicity, and age groups.

	**Version 1**			**Version 2**			**Version 3**		
	**Gender**	**Ethnicity**	**Age**	**Gender**	**Ethnicity**	**Age**	**Gender**	**Ethnicity**	**Age**
Item 1	0.07	−0.03	−0.07	0.11	−0.10	−0.07	0.14	−0.12	0.18^**^
Item 2	0.17^***^	0.00	−0.07^*^	0.25^***^	0.06	−0.09	0.35^***^	0.12	0.15^*^
Item 3	−0.12^*^	0.07	0.46^***^	−0.17^*^	0.11	0.68^***^			
Item 4	−0.08	0.04	0.56^***^	−0.12	0.11	0.86^***^			
Item 5	0.11^*^	0.00	0.23^***^	0.14	0.00	0.30^***^			
Item 6	0.00	0.06	−0.21^***^	0.00	0.08	−0.22^**^	0.00	0.16	−0.03
Item 7	−0.12^*^	−0.07	−0.14^**^	−0.16^*^	−0.07	−0.15^*^	−0.23^**^	−0.07	0.06
Item 8	0.00	−0.12	−0.18^***^	0.00	−0.14	−0.20^**^	0.00	−0.16	0.00
Item 9	−0.09	0.00	−0.31^***^	−0.10	0.00	−0.32^***^	−0.17	0.03	−0.19^*^
Item 10	−0.06	0.00	−0.29^***^	−0.08	0.00	−0.36^***^	−0.17	0.00	−0.24^**^

*gender (boys vs. girls); ethnicity (majority vs. minorities); age (8–12 vs. 13–18); DIF contrast values were reported;*

* *p < 0.05*,

** *p < 0.01*,

*** *p < 0.001*.

### Known-Group Validity

Differences in the KIDSCREEN scores by socioeconomic status, health status, and academic performance are shown in Table 5. The scores of the three versions exhibited a significant small effect size among participants with different socioeconomic statuses (η^2^ ranged from 0.007 to 0.017) or with different academic performance (η^2^ ranged from 0.041 to 0.058). However, the effect size on health status was moderate in version 3 (η^2^ = 0.073).

**Table 5:** Known-group validity tests.

	**Version 1**		**Version 2**		**Version 3**	
	**η^2^**	**F-value**	**η^2^**	**F-value**	**η^2^**	**F-value**
Socioeconomic status	0.007	3.25^*^	0.009	4.21^**^	0.017	7.79^***^
Health status	0.022	10.40^***^	0.037	17.72^***^	0.073	36.01^***^
Academic performance	0.041	19.43^***^	0.046	22.06^***^	0.058	27.92^***^

*eta square (η^2^);*

* *p < 0.05*,

** *p < 0.01*,

*** *p < 0.001*.

### Concurrent Validity

The Pearson correlation coefficients between the version 1 and the BMSLSS, and between the version 2 and the BMSLSS, exhibited a low coefficient effect size (0.26 for version 1; 0.28 for version 2). However, the coefficient (0.37) was moderate for the correlation between the version 3 and the BMSLSS.

## Discussion

This was the first study to examine the psychometric properties of KIDSCREEN-10 in the context of Chinese society. First, results found that the internal consistency of the questionnaire was acceptable, which were consistent with prior studies on KIDSCREEN-10 (Erhart et al., 2009; Ravens-Sieberer et al., 2010; Stevanovic et al., 2013; Nezu et al., 2016). Second, known-group validity was verified. The known-group validity, also named as construct validity, reflects that a test can discriminate between two groups known to vary on the variables of interest (Langevin, 2009; Hendriks et al., 2017). In this study, the HRQoL level measured by the KIDSCREEN-10 questionnaire demonstrated significant differences among different socioeconomic statuses/health statuses/academic performance groups. Similar differences across socioeconomic status were also found in studies from Turkey (Baydur et al., 2016) and European countries (Erhart et al., 2009; Ravens-Sieberer et al., 2010). Moreover, KIDSCREEN-10 has validity in predicting HRQoL levels of children and adolescents.

Seven items (i.e., items 1, 2, 6, 7, 8, 9, and 10) exhibited ceiling effects. Although previous studies did not exhibit such effects, these studies examined the overall ceiling effect of the questionnaire rather than each item (Nik-Azin et al., 2014; Baydur et al., 2016). The ceiling effects indicate that most of the items were easy for the respondents, and thus the KIDSCREEN-10 cannot differentiate well the respondents with high degrees of HRQoL. However, these items do not demonstrate floor effects. This is meaningful from a public health and clinical perspective because it is more important to differentiate well between respondents with low-QoL as these are of risks for various health problems and represent the target population for interventions.

The original KIDSCREEN-10 questionnaire measured by a 5-point Likert scale (i.e., response categories include “never,” “seldom,” “sometimes,” “usually,” and “always”) demonstrated disordered response categories. It is because response options with similar meaning may make it difficult for respondents to distinguish the differences of options when answering, and thereby result in disordered categories (Zhong et al., 2014). After collapsing the categories of “seldom” and “sometimes,” the response categories ordered appropriately. Therefore, the KIDSCREEN-10 questionnaire was better to be measured by the four response categories in China.

Inconsistent with prior finding that all items of KIDSCREEN-10 in 15 European countries exhibited a good fit to the Rasch partial credit model (Erhart et al., 2009), this study found that item 3 (“Have you felt sad?”) and item 4 (“Have you felt lonely?”) demonstrated misfit to the model. No matter before or after merging the response categories, both infit and outfit MNSQ values of items 3 and 4 were higher than the acceptable cut-off value of 1.4 (Zhong et al., 2014). Items misfit indicated that the response of these items was inconsistent with the overall response pattern (Liu et al., 2016). This might indicate that Chinese school-aged children's cognition of mental health was different from other aspects (such as activities participation, peer relationships, school performance, etc.) measured by KIDSCREEN-10 since these two items measured the mental health of a child (i.e., depressive moods and emotions and stressful feelings) (Ravens-Sieberer et al., 2010). Meanwhile, this result might explain why the eigenvalue of the first contrast of the original KIDSCREEN-10 was higher than 2 thus violating the assumption of unidimensionality. Moreover, after removing these two items and reconducting the Rasch analysis, we found item 5 (“Have enough time for self?”) was also a misfit item. Finally, the seven-item questionnaire assessed by a four-point Likert scale fitted the model well. It should be pointed out that, unlike deleting items 3 and 4 would delete a dimension (i.e., mental health) of the questionnaire, although item 5 was removed as well, the dimensions of the seven-item version did not decrease further. Because the seven-item questionnaire includes item 6, which reflects the same dimension (i.e., autonomy) as item 5 (Ravens-Sieberer et al., 2008, Ravens-Sieberer et al., 2014; Nezu et al., 2016).

Although the previous Rasch analyses showed the KIDSCREEN-10 was valid and reliable (Erhart et al., 2009; Ravens-Sieberer et al., 2014), they did not perform the DIF analysis. In the current study, both item 3 and item 4 exhibited DIF when comparing respondents aged from 8 to 11 years and 12 to 20 years. It can be implied that the use of the KIDSCREEN-10 in China was difficult because these items (items 3 and 4) cannot measure the HRQoL level of children and adolescents independently of age. Therefore, this study suggested that, to avoid the questionable conclusion, the DIF analysis should be conducted when the Rasch method is employed.

In summary, in contrast with previous studies that found the original KIDSCREEN-10 had acceptable validity and reliability in European countries by using the Rasch measurement model (Erhart et al., 2009; Ravens-Sieberer et al., 2014), our results showed that the psychometric features of the original KIDSCREEN-10 were deficiencies. This may be due to cultural differences. This study was conducted in the Chinese Mainland. China has a collectivist culture, which is different from the individualist culture of European countries. The psychometric properties of the seven-item version measured with four response categories (“never,” “seldom/sometimes,” “usually,” and “always”) performed better than the original KIDSCREEN-10, but it should be noted that the seven-item version does not contain items (i.e., items 3 and 4) on psychological well-being (Robitail et al., 2007), and thus it is not appropriate to measure the general HRQoL using the seven-item questionnaire. Accordingly, instead of advocating the seven-item version, this paper suggests that the psychological properties of the KIDSCREEN-10 should be further tested by using CTT. Previous studies demonstrated that KIDSCREEN-10 provided a CTT reliable and valuable assessment of general HRQoL (Erhart et al., 2009; Stevanovic et al., 2013; Nik-Azin et al., 2014; Baydur et al., 2016; Nezu et al., 2016). Moreover, the KIDSCREEN-52, the parent version of the KIDSCREEN-10, has been validated by CTT in China (Ng et al., 2015; Zhu et al., 2019); thus, the KIDSCREEN-10 may not permit a Rasch-based measurement of general HRQoL, but fulfill the requirements of CTT.

This study has some limitations. First, the values of infits and outfits calculated by using the WINSTEPS software may lead to a high type I error rate because the software only calculates unconditional outfit and infit statistics, the results may become unreliable for sample sizes above 250 (Müller, 2020). Second, our sample, investigated from an autonomous county for ethnic minorities where the population of minorities was more than the national majority, may result in selection bias. Third, the concurrent validity of KIDSCREEN was examined by using the Pearson correlation coefficient. This method may under- or overestimate the predictive power, since it cannot adjust for confounders. Moreover, this study cannot provide the test–retest reliability due to the cross-sectional investigation.

## Conclusions

The Mandarin version of the KIDSCREEN-10 did not perform good psychometrical properties in China by using the Rasch analysis. The KIDSCREEN-10 demonstrated disordered response categories, item misfit, unidimensionality, and DIF. After adjusting the response categories and removing the three misfit items, the seven-item questionnaire measured by the four response categories perform good measurement characteristics. However, the seven-item version was not appropriate to measure general HRQOL because it did not contain items on psychological well-being of HRQoL. Therefore, instead of advocating the seven-item version, this paper suggested that the psychological properties of the KIDSCREEN-10 should be further tested by using CTT.

## Data Availability Statement

The raw data supporting the conclusions of this article will be made available by the authors, without undue reservation.

## Ethics Statement

The studies involving human participants were reviewed and approved by Institutional review board of Sichuan University (K2019067). Written informed consent to participate in this study was provided by the participants' legal guardian/next of kin.

## Author Contributions

ZG and YL proposed and designed this study. ZH collected the data. ZG and ZH analyzed the data and wrote the first draft. YL and JX made a revision. All authors read and approved the final manuscript.

## Conflict of Interest

The authors declare that the research was conducted in the absence of any commercial or financial relationships that could be construed as a potential conflict of interest.

## Publisher's Note

All claims expressed in this article are solely those of the authors and do not necessarily represent those of their affiliated organizations, or those of the publisher, the editors and the reviewers. Any product that may be evaluated in this article, or claim that may be made by its manufacturer, is not guaranteed or endorsed by the publisher.
