# Supplementary material for: Validation of the Chinese Version of KIDSCREEN-10 Quality of Life Questionnaire: A Rasch Model Estimation
Source: Front Psychol. 2021 Aug 16;12:647692. doi: 10.3389/fpsyg.2021.647692 (PMC8415151; doi:10.3389/fpsyg.2021.647692)
Supplement: Supplementary file 1 [file Table_1.DOCX]

Supplementary Material

# Supplementary Table

Table 1 KIDSCREEN-10 scale and its Chinese version

| Items | Chinese translation |
| --- | --- |
| Item 1: Have you felt fit and well? | 1．你感到身体状态良好吗? |
| Item 2: Have you felt full of energy? | 2．你感到精力充沛吗？ |
| Item 3: Have you felt sad? | 3．你感到悲伤吗？ |
| Item 4: Have you felt lonely? | 4．你感到孤独吗？ |
| Item 5: Have you had enough time for yourself? | 5．你有足够的时间给自己吗？ |
| Item 6: Have you been able to do the things that you want to do in your free time? | 6．你在有空时能做自己想做的事情吗？ |
| Item 7: Have your parent(s) treated you fairly? | 7．父母对你公平吗？ |
| Item 8: Have you had fun with your friends? | 8．你跟朋友玩得开心吗？ |
| Item 9: Have you got on well at school? | 9．你在学校的成绩理想吗？ |
| Item 10: Have you been able to pay attention? | 10．你能专心上课吗？ |

Note: our recommended response categories are “never (从不)”, “sometimes (有时)”, “often (经常)”, “always (总是)”. We recommend to drop the item three and item four because they exhibit misfit to the Rasch model.
